# Development and validation of a preoperative MRI-based radiomics nomogram to predict progression-free survival in patients with clival chordomas

**DOI:** 10.3389/fonc.2022.996262

**Published:** 2022-12-16

**Authors:** Yixuan Zhai, Jiwei Bai, Yake Xue, Mingxuan Li, Wenbin Mao, Xuezhi Zhang, Yazhuo Zhang

**Affiliations:** ^1^ Beijing Neurosurgical Institute, Capital Medical University, Beijing, China; ^2^ Department of Neurosurgery, The First Affiliated Hospital of Zhengzhou University, Zhengzhou, China; ^3^ Department of Neurosurgery, Beijing Tiantan Hospital, Capital Medical University, Beijing, China; ^4^ Center of Brain Tumor, Beijing Institute for Brain Disorders, Capital Medical University, Beijing, China; ^5^ China National Clinical Research Center for Neurological Diseases, Beijing, China

**Keywords:** radiomics, nomogram, chordoma, prognosis, prediction

## Abstract

**Objectives:**

The aim of this study was to establish and validate a MRI-based radiomics nomogram to predict progression-free survival (PFS) of clival chordoma.

**Methods:**

A total of 174 patients were enrolled in the study (train cohort: 121 cases, test cohort: 53 cases). Radiomic features were extracted from multiparametric MRIs. Intraclass correlation coefficient analysis and a Lasso and Elastic-Net regularized generalized linear model were used for feature selection. Then, a nomogram was established *via* univariate and multivariate Cox regression analysis in the train cohort. The performance of this nomogram was assessed by area under curve (AUC) and calibration curve.

**Results:**

A total of 3318 radiomic features were extracted from each patient, of which 2563 radiomic features were stable features. After feature selection, seven radiomic features were selected. Cox regression analysis revealed that 2 clinical factors (degree of resection, and presence or absence of primary chordoma) and 4 radiomic features were independent prognostic factors. The AUC of the established nomogram was 0.747, 0.807, and 0.904 for PFS prediction at 1, 3, and 5 years in the train cohort, respectively, compared with 0.582, 0.852, and 0.914 in the test cohort. Calibration and risk score stratified survival curves were satisfactory in the train and test cohort.

**Conclusions:**

The presented nomogram demonstrated a favorable predictive accuracy of PFS, which provided a novel tool to predict prognosis and risk stratification. Our results suggest that radiomic analysis can effectively help neurosurgeons perform individualized evaluations of patients with clival chordomas.

## Introduction

Chordoma is a rare malignant neoplasm that arises from the remnants of the primitive notochord (1). The annual incidence is lower than one case per million people (2). Chordoma mainly involves the axial skeleton, and about 25% of chordoma originates from the skull base (3). Skull base chordoma has the tendency to invade surrounding structures which makes it difficult to achieve gross-total resection (GTR) in the operation (4). Furthermore, chordoma isn’t sensitive to chemotherapy and radiotherapy, resulting in extensive recurrences. The local recurrence rate is as high as 50% in four years after surgery. Until now, there is no effective method for predicting recurrence (5).

Radiomics generally refers to the extraction and analysis of large amounts of advanced quantitative imaging features with high throughput from medical images obtained using CT, MRI or PET. The researchers are able to develop models that may potentially improve diagnostic, prognostic, and predictive accuracy. Radiomics has been widely used in prediction of tumor consistency, prognosis, and blood supply in many tumors such as pituitary adenomas (6), gliomas (7), meningiomas (8) et al. It enables doctors to deliver more personalized treatment with regard to tumor detection, prediction of tumor subtypes, and prognosis. However, few studies have focused on radiomics application in chordoma. Therefore, in this study, we aimed to establish a radiomics nomogram to predict progression-free survival (PFS) in patients with clival chordomas.

## Methods

### Patients

Our study retrospectively analyzed skull base chordoma patients who received operations at Beijing Tiantan Hospital from September 2003 to September 2014. The inclusion criteria were as follows: 1) chordoma diagnosis was confirmed by the pathological report, 2) medical records were complete, and 3) lesions were located in the clival region. Exclusion criteria were as follows: 1) incomplete medical records, 2) poor quality imaging, and 3) lack of patient follow-up. Accordingly, 174 skull base chordoma patients were included in the study.

The patients were divided randomly into train cohort (n=121) and test cohort (n=53). Patients’ data was collected, including gender, age, course of disease, chief complaint, KPS score, degree of resection, postoperative treatment, and complications. The gross total resection (GTR) was defined as no residual tumor in the post-operative image. Non-gross total resection (NGTR) was defined as residual tumor detected in the post-operative image.

### MRI acquisition

All patients underwent MR imaging scan before operation. Three clinical scanners (Siemens Healthcare, Toshiba Medical Systems, and GE Medical Systems) were used in the study. The MR imaging protocol included T1-weighted contrast-enhanced imaging with fluid-attenuated inversion-recovery sequence (T1C), T1-weighted imaging with fluid-attenuated inversion-recovery sequence (T1WI), and T2-weighted imaging sequence (T2WI). The T1C sequence was acquired with the following parameters: Repetition time (TR):2031.01-2803.86ms, Echo time (TE): 10.35-19.76ms, Flip angle: 90°, and Slice thickness: 4-6.5mm. The T1WI sequence was acquired with the following parameters: TR: 2031.01-2458.22ms, TE:9.50-19.76ms, Flip angle: 90°, and Slice thickness: 5-6.5mm. The T2WI sequence was acquired with the following parameters: TR:2680-6000ms, TE:102.17-123.62ms, Flip angle: 90°, and Slice thickness: 5-6.5mm.

### Tumor segmentation, preprocessing, and feature extraction

The tumors were manually drawn on T1C imaging by two neuroradiologists independently, using a free 3D-Slicer software (v4.9.0).

Preprocessing was performed by SimpleITK package (v1.2.4). First, image registration was performed to register T1, and T2 sequence images to the T1C sequence images. Next, N4 bias field correction was applied to each sequence images to correct non-uniformities in intensity. The feature extraction was performed using PyRadiomics package (v3.0). The detail parameter settings are provided in the Supplementary document. For each imaging sequence, a total of 1106 radiomics features were calculated. For each patient, a total of three imaging sequences were calculated, which generated 3318 radiomic features.

### Radiomic feature selection

To avoid overfitting, feature selection was performed before model training. Features were selected by two steps. First, the ROIs drawn by the two neuroradiologists were compared, and intraclass correlation coefficients (ICCs) were calculated. If the ICCs > 0.7, the radiomic features were defined as stable features. Then, the stable features were analyzed by a Lasso and Elastic-Net regularized generalized linear model (glmnet). Five-fold cross-validation with minimizing deviance was performed to find the optimal λ. With the optimal λ, the coefficient of each feature was calculated, then features with non-zero coefficients were selected. This step was repeated 1000 times. The features that appeared more than 150 times were selected as the prognostic radiomic features.

### Survival model training and nomogram establishment

Cox regression analysis was used to identify the prognostic factors. First, prognostic radiomics features and all the clinical variables were analyzed by univariate Cox regression. Then, all the variables with *p* value < 0.1 were further analyzed by multivariate Cox regression with the backward model (9). After that, all the significant variables were identified as the independent prognostic variables.

The Rad-score for each patient was calculated using the sum of the values of selected radiomic features weighted by the corresponding mean non-zero coefficients (see Supplementary document). The nomogram was established with the Rad-score and the independent prognostic clinical variables.

### Predictive performance of nomogram

**Figure 1:**
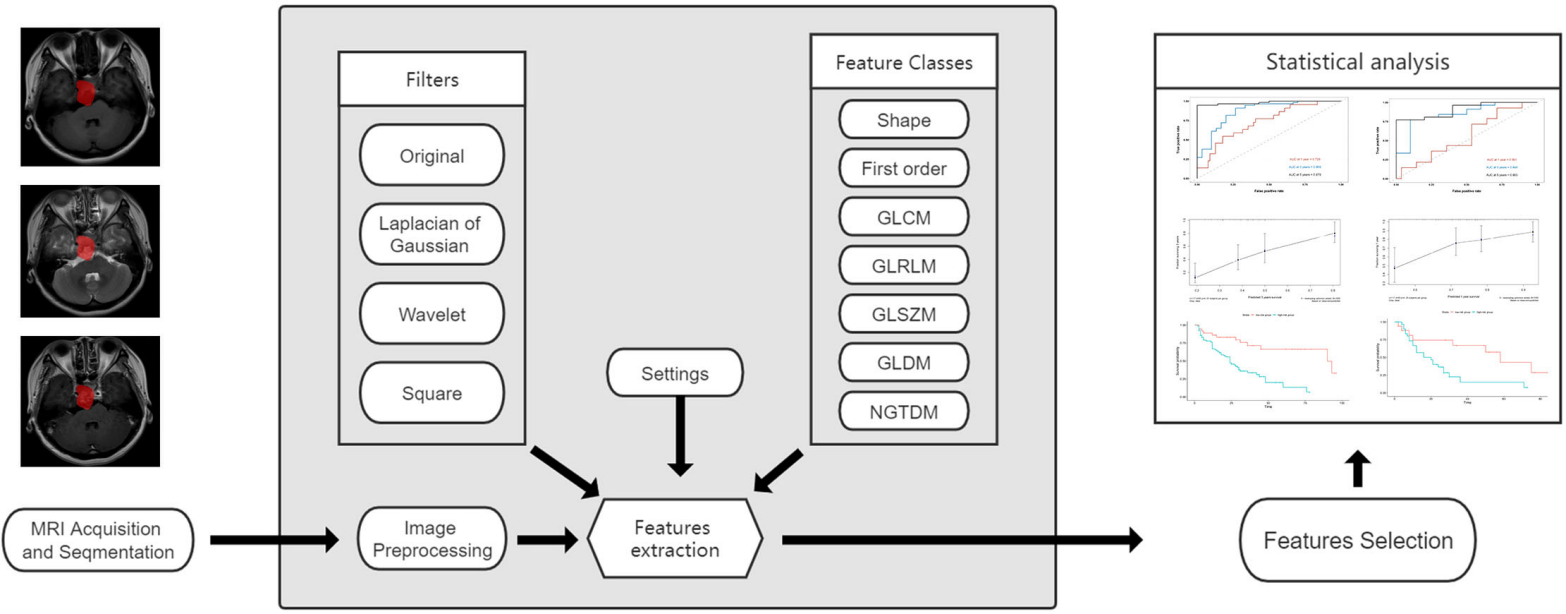
The flow chart of the study.

To evaluate the nomogram performance, the area under curve (AUC) of receiver operating characteristic (ROC) was performed in the train and test cohorts. A value of AUC > 0.7 indicates a good discrimination. Then, to compare the consistency between the predicted value and actual value, a calibration curve was drawn (10). Lastly, we calculated the risk score for each patient according to the nomogram. Using the risk score, patients were divided into two groups (low-risk and high-risk). If the risk score was lower than the mean score of the train cohort, the patient was placed in the low-risk group, otherwise they were placed in the high-risk group. The PFS was compared between the low-risk and high-risk group using the Kaplan-Meier method. And Rad-scores were also compared between the two groups. The flowchart of this study is shown in **Figure 1**.

### Statistical analysis

Statistical analysis was conducted in Python (v3.7.6) with the packages (“os”, “pandas”, “SimpleITK”, “pyradiomics”) and R software (v4.0.0) with the packages (“survival”, “rms”, “plyr”, “glmnet”, “timeROC”, “survminer”).

## Results

### Patient clinical characteristics

A total of 174 patients (97 male and 77 female) were included in the study. There are 140 primary chordomas and 34 recurrent chordomas. The mean age was 40.51 years old (range: 8-78 years old), the mean course of disease was 21.77 months (range: 1-360 months). The KPS score ranged from 50 to 90 (mean: 80). GTR was achieved in 48 patients, and NGTR was achieved in 126 patients. There were 65 patients (37.4%) receiving post-operative radiotherapy. The total radiotherapy dose was between 50 Gy and 64 Gy (mean: 54.1 Gy). The mean follow-up interval was 86 months (range: 52-185 months). Patient characteristics are shown in **Table 1**, which were similar overall between the train and test cohorts.

**Table 1:** Patient characteristics.

Characteristics	Train cohort	Test cohort	*P* value
No. of patients	121	53	
Gender, n			
Male	72	25	0.18
Female	49	28	
Mean age (range), yrs	40.72 (11-78)	40.04 (8- 63)	0.77
Mean course of disease (range), mos	17.62 (1-240)	31.20 (1-360)	0.15
Primary chordoma, n	98	42	0.95
Recurrent chordoma, n	23	11	
Degree of resection, n			
GTR	34	14	0.96
NGTR	87	39	
Post-operative radiotherapy, n			
Yes	40	25	0.11
No	81	28	
Complications, n			
Yes	29	16	0.5
No	92	37	

GTR, gross total resection; NGTR, non-gross total resection.

### Radiomic feature selection

In total, 3318 radiomic features were extracted, and 2563 radiomic features were selected as stable features by ICCs (see **Supplementary Table 1**). Through Lasso and Elastic-Net regularized generalized linear model selection, seven radiomic features were selected.

### Survival model training and nomogram establishment

Univariate Cox regression analysis was performed to detect the relationship between PFS and each of the seven radiomic features and clinical factors. Among the 7 radiomic features, 4 features were favorable prognostic features. Among the 10 clinical factors, 2 factors (GTR and primary chordoma) were favorable clinical factors. Multivariate Cox regression analysis was further performed. We found that 2 clinical factors and 4 radiomic features were significant prognostic factors (see **Table 2**).

**Table 2:** Univariate and multivariate Cox regression analysis.

Variables	Univariate analysis			Multivariate analysis		
	HR	95%CI	*P value*	HR	95%CI	*P value*
Clinical factors						
Age	1.01	0.99-1.02	0.3653			
Gender (female vs male)	1.14	0.74-1.76	0.5617			
Course of disease	1	0.99-1	0.2571			
KPS score	0.99	0.96-1.02	0.4817			
Pituitary gland involved	1.04	0.67-1.6	0.8626			
Brainstem involved	1.14	0.71-1.85	0.5908			
Degree of resection (NGTR vs GTR)	5.05	2.71-9.43	<0.001	4.69	2.50-8.83	<0.001
Recurrent vs primary chordoma	2.35	1.41-3.92	0.001	1.77	1.06-2.96	0.03
With vs without post-operative radiotherapy	1.48	0.96-2.29	0.0757			
With vs without complication	1.04	0.64-1.72	0.8638			
Radiomics features						
T1_square_glrlm_ShortRunEmphasis	1.79	1.38-2.31	<0.001			
T1_wavelet.HLL_firstorder_Range	1.65	1.32-2.07	<0.001			
T1_wavelet.HLL_ngtdm_Complexity	1.66	1.33-2.08	<0.001	1.32	1.03-1.69	0.03
T1c_square_gldm_LowGrayLevelEmphasis	0.5	0.38-0.67	<0.001	0.57	0.41-0.78	<0.001
T1c_wavelet.HHH_firstorder_Range	1.47	1.18-1.82	<0.001	1.14	1.10-1.79	0.006
T2_wavelet.HLL_firstorder_Mean	1.89	1.44-2.48	<0.001	1.44	1.06-1.97	0.02
T2_square_firstorder_Median	0.55	0.42-0.71	<0.001			

CI, confidence interval; GTR, gross total resection; NGTR, non-gross total resection; HR, hazard ratio.

**Figure 2:**
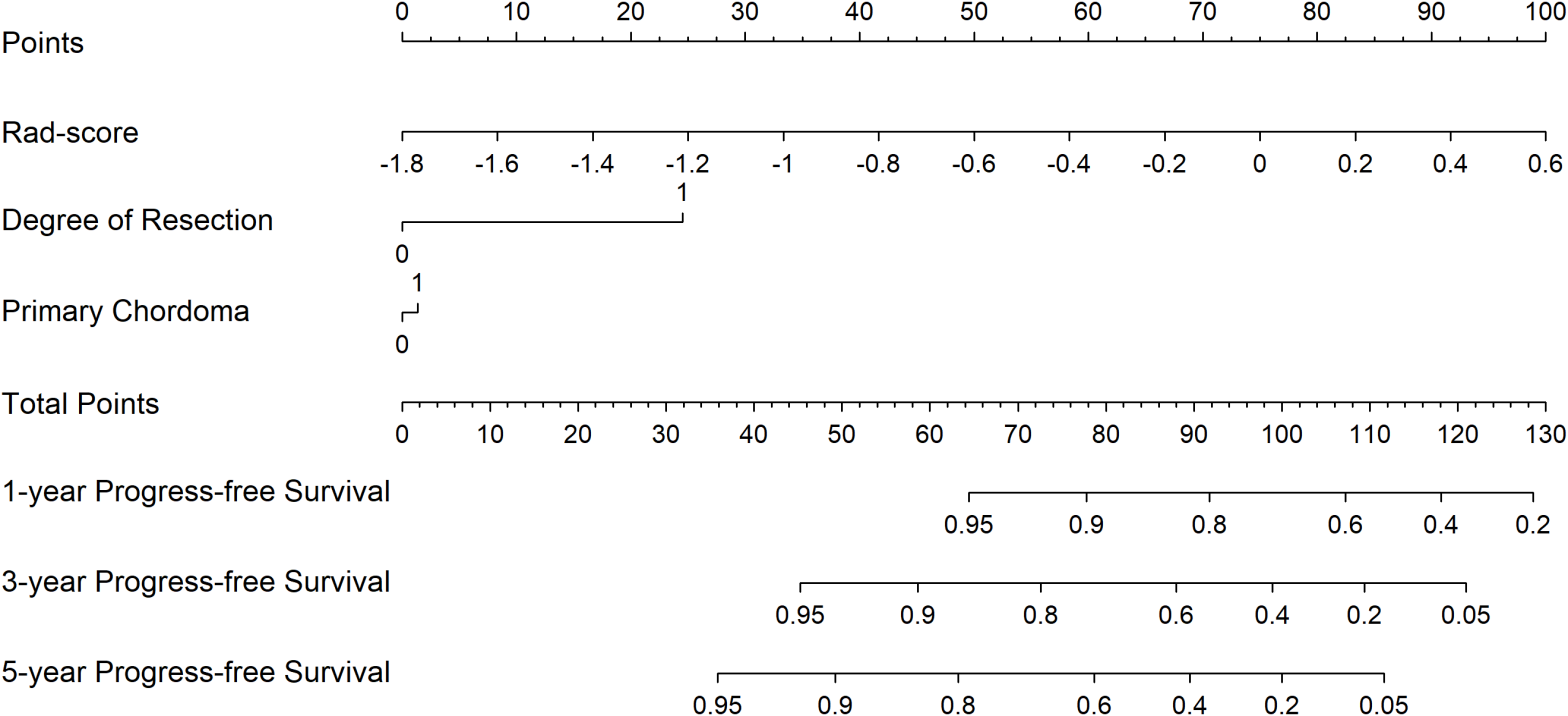
Radiomics nomogram for PFS prediction.

The Rad-score for each patient was calculated in train and test cohorts. The formula of Rad-score is presented in the Supplementary Document. Based on the clinical factors and Rad-scores, we established a nomogram for easier clinical use (**Figure 2**).

For each patient, prognostic points were calculated by the following equation: Total points = 41.67 × (Rad-score + 1.8) + 1.39 × (primary chordoma or not) + 24.51 × (degree of resection). Higher total points indicated worse prognosis. The established 1-, 3-, and 5-year PFS rates were obtained according to the total points.

**Figure 3:**
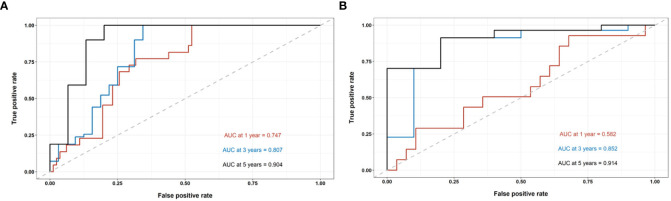
The performance evaluation of the radiomics nomogram. **(A)** The ROC curve in the train cohort; **(B)** The ROC curve in the test cohort.

**Figure 4:**
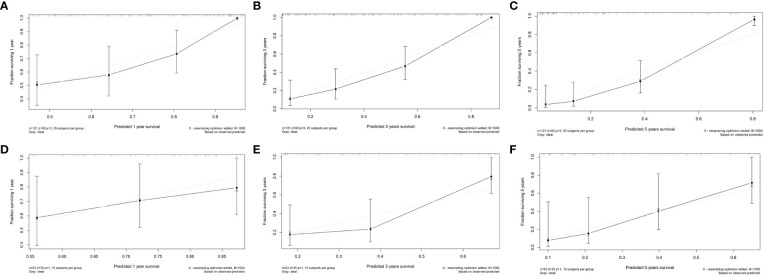
The calibration curves of the radiomics nomogram. **(A)** Calibration curve for prediction 1-year PFS in the train cohort; **(B)** Calibration curve for prediction 3-year PFS in the train cohort; **(C)** Calibration curve for prediction of 5-year PFS in the train cohort; **(D)** Calibration curve for prediction of 1-year PFS in the test cohort; **(E)** Calibration curve for prediction of 3-year PFS in the test cohort; **(F)** Calibration curve for prediction of 5-year PFS in the test cohort.

In the train cohort, the AUCs of the model were 0.747, 0.807 and 0.904 for PFS prediction at 1, 3 and 5 years, respectively (see **Figure 3**). This showed that the model had a satisfactory predictive ability. The calibration curves were drawn to test the model consistency which showed the nomogram had a satisfactory calibration curve (see **Figure 4**).

### Model validation and comparison

The AUCs of the model in the test cohort were 0.582, 0.852, and 0.914 for PFS prediction at 1, 3 and 5 years, respectively. This demonstrated a satisfactory prediction performance (see **Figure 3**). The calibration curves of the test cohort also showed a satisfactory calibration (see **Figure 4**).

**Figure 5:**
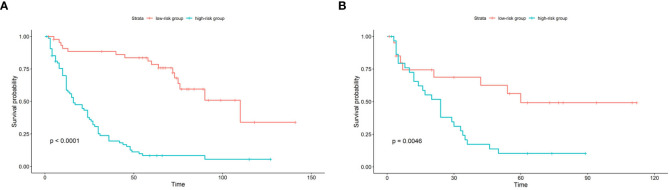
Kaplan-Meier survival curve of patients in the train cohort **(A)** and test cohort **(B)** stratified by nomogram predicted score. In the low-risk group, the score is lower than the mean score of the group; in the high-risk group, the score is higher than the mean score of the group.

According to the established nomogram, the risk score of each patient was calculated. The mean risk score of the train cohort was 91.98 (range: 29.82-122.44). The mean risk score of the test cohort was 95.40 (range: 23.17-125.45). Patients were divided into two groups by the mean risk score of the train cohort. If the risk score was lower than the mean score of train cohort, the patient was placed in the low-risk group, otherwise they were placed in the high-risk group. In the train cohort, the median PFS of the low-risk group was 110 months, and the median PFS of the high-risk group was 16 months (p<0.0001). In the test cohort, the median PFS of the low-risk group was 60 months, and the median PFS of the high-risk group was 24 months (p=0.0046) (see **Figure 5**).

## Discussion

The chordoma has the tendency of recurrence which results in poor prognosis. It has been reported that the 5-year PFS rate is as low as 59% (11). It has been reported that many factors are related to prognosis, such as total resection (12), post-operative radiotherapy (13), PBRM1 (14), PARP1, PDGFR-β (15), etc. A few prediction models were established using these prognostic factors. However, the performance of these models was not satisfactory. Therefore, we established a more efficient prediction model in this study.

Many studies have reported clinical factors that could predict patient PFS, but no consensus has been reached. In our study, we found that degree of resection was the independent prognostic factor. Radical resection can significantly prolong patient PFS. Sekhar et al (16) retrospectively studied 42 patients and built a Chordoma Grading System. In these patients, complete resection rate was 36%, and subtotal resection rate was 64%. After Cox proportional hazards model analysis, they found that complete resection rate was significantly associated with PFS. Carl H Snyderman et al (17) present their endoscopic endonasal experience in the treatment of 60 patients with cranial base chordomas. The overall rate of GTR was as high as 66.7%. The mean PFS was 14.4 months. The recurrence rate of patients who underwent GTR was 20%, which was significantly lower than that of patients who underwent NGTR (60%). The high GTR rate can be attributed to the extensive experience of the surgeons. In most published studies, the GTR rate was about 30% to 50%. Thus, improvement of operative technique was the key to improve GTR rate. In this study, we also found that the PFS of primary chordoma was significantly longer than that of recurrent chordoma, which emphasized the importance of the first operation. If GTR is achieved in the primary chordoma, the prognosis will be significantly improved. Recently, some molecular features have been reported to be important prognostic factors in clival chordomas. Georgios A Zenonos et al (18) prospectively evaluated the 1p36 and 9p21 state, Ki-67 expression level in 105 clival chordomas samples, and found that homozygous 1p36 deletions and 9p21 deletions were independent prognostic factors.

Radiomics is a new area of study, which has been widely used in the differential diagnosis and prognosis prediction of many tumors (19–21). Nan Hong et al (22) developed a clinical-radiomics nomogram to differentiate sacral chordoma and sacral giant cell tumor before operation, which yielded an AUC of 0.948. Jie Tian et al (23) established a radiomic signature with multiparametric MRI for differentiating skull base chordoma and chondrosarcoma, which yielded an AUC of 0.975 and 0.872 in the train and test cohorts, respectively.

Only two articles that used radiomic features to predict prognosis of patients with clival chordoma have been published. Jie Tian et al (24) enrolled 80 patients with skull base chordomas, and selected 5 features from MR images to build a radiomics signature, which was used to train a logistic regression model. The model achieved an AUC=0.8600 in the train cohort and AUC=0.8568 in the test cohort. One year later, the author (25) developed a clinical-radiomic model to predict PFS of chordoma. They enrolled 148 patients, including 64 with disease progression. The Harrell’s concordance index of their model was only 0.745 for the test cohort. In our study, we enrolled more patients with clival chordoma and extended follow-up time. Thus, our model achieved higher AUC and accuracy. The AUCs of our model were as high as 0.904 and 0.914 for PFS prediction at 5 years in the train and test cohorts, respectively. In addition, our model displayed a good calibration and discrimination ability. With the help of a nomogram, a neurosurgeon can stratify their patients. If the patient is classified in the high-risk group, the surgeon could shorten the follow-up interval and use more therapeutic measures. Thus, it is possible to develop an individualized treatment plan.

Our study has several limitations. First, all the patients were enrolled over the course of several years from one center, which may lead to patient selection bias. A multicenter study is needed for further analysis. Second, patient imaging was acquired by different scanners, which may increase the data heterogeneity bias. To avoid this, all imaging was subjected to imaging normalization before feature extraction.

## Conclusion

In our study, we developed and validated a nomogram based on the multiparametric MRI radiomics signatures and clinical factors. The nomogram demonstrated a favorable predictive accuracy of PFS which provided a novel tool to predict prognosis and risk stratification. Our results suggest that radiomics analysis can effectively help neurosurgeons perform individualized evaluations of patients with clival chordomas.

## Data availability statement

The raw data supporting the conclusions of this article will be made available by the authors, without undue reservation.

## Author contributions

YXZ and YZZ designed the study. JB and ML collected clinical and MRI data. YX, WM, and XZ pre-processed patients MR imaging and drew the ROI. YXZ and JB analyzed the data and developed prediction model. YXZ wrote the manuscript. All authors contributed to the article and approved the submitted version.
