# Supplementary material for: Development and validation of a preoperative MRI-based radiomics nomogram to predict progression-free survival in patients with clival chordomas
Source: Front Oncol. 2022 Dec 16;12:996262. doi: 10.3389/fonc.2022.996262 (PMC9800789; doi:10.3389/fonc.2022.996262)
Supplement: Supplementary file 2 [file DataSheet_1.docx]

**Data Supplement S1: detail parameter settings of feature extraction in PyRadiomics package.**

imageType:

Original: {}

LoG:

sigma: [3.0, 5.0]

Wavelet: {}

Square: {}

featureClass:

shape:

firstorder:

glcm:

- 'Autocorrelation'

- 'JointAverage'

- 'ClusterProminence'

- 'ClusterShade'

- 'ClusterTendency'

- 'Contrast'

- 'Correlation'

- 'DifferenceAverage'

- 'DifferenceEntropy'

- 'DifferenceVariance'

- 'JointEnergy'

- 'JointEntropy'

- 'Imc1'

- 'Imc2'

- 'Idm'

- 'Idmn'

- 'Id'

- 'Idn'

- 'InverseVariance'

- 'MaximumProbability'

- 'SumEntropy'

- 'SumSquares'

glrlm:

glszm:

gldm:

ngtdm:

setting:

normalize: true

normalizeScale: 100

interpolator: 'sitkBSpline'

resampledPixelSpacing: [3, 3, 3]

binWidth: 5

voxelArrayShift: 300

label: 1

**Data Supplement S2: Rad-score calculation formula**

Rad-score = T1_wavelet.HLL_firstorder_Range*(0.05490523)+

T1c_square_gldm_LowGrayLevelEmphasis*(-0.264439)+

T1c_wavelet.HHH_firstorder_Range*(0.09653172)+

T2_wavelet.HLL_firstorder_Mean*(0.1115917)
